# Long-Acting β_2_ Adrenergic Receptor Agonist Ameliorates Imiquimod-Induced Psoriasis-Like Skin Lesion by Regulating Keratinocyte Proliferation and Apoptosis

**DOI:** 10.3389/fphar.2022.865715

**Published:** 2022-06-20

**Authors:** Rui Xu, Shi Feng, Zhou Ao, Yingxiang Chen, Congping Su, Xiuling Feng, Qin Fu, Xiaoyan Yang

**Affiliations:** ^1^ Department of Pharmacology, School of Basic Medicine, Tongji Medical College, Huazhong University of Science and Technology, Wuhan, China; ^2^ The Key Laboratory for Drug Target Researches and Pharmacodynamic Evaluation of Hubei Province, Wuhan, China

**Keywords:** psoriasis, cAMP, PKA, PDE4 inhibitor, β_2_ adrenergic receptor agonist, ERK

## Abstract

Psoriasis is a chronic inflammatory disease that affects approximately 1%–5% of the population worldwide. Considering frequent relapse, adverse drug reactions, and large costs of treatment, it is urgent to identify new medications for psoriasis. Keratinocytes play an essential role during psoriasis development, and they express high levels of β_2_-Adrenergic receptor (β_2_-AR), which increases intracellular cAMP levels when activated. Increased level of cAMP is associated with the inhibition of epidermal cell proliferation. In the present study, we observed the effect of salmeterol, a long-acting β_2_-AR agonist, on the proliferation and apoptosis of keratinocytes as well as imiquimod-induced psoriasis-like skin lesions in mice. As phosphodiesterase 4 (PDE4) inhibitors increases intracellular cAMP concentration by inhibiting its inactivation, we further explored the synergetic effect of a PDE4 inhibitor and salmeterol on psoriasis-like skin lesions in mice. Our results indicated that salmeterol effectively inhibited the proliferation of HaCaT cells induced by TNF-α and serum, and this effect was accompanied by significantly increased apoptosis and CREB phosphorylation, which were reversed by the PKA inhibitor, H89. Salmeterol ameliorated imiquimod-induced psoriasis-like skin lesions in mice, but salmeterol combined with a PDE4 inhibitor had no synergetic effect in improving skin lesions in mice. Of note, the synergistic effects of anti-proliferation and induction of apoptosis in HaCaT cells appeared by inhibiting ERK signaling. In summary, salmeterol, a long-acting β_2_-AR agonist, alleviates the severity of psoriasis *via* inhibiting the proliferation and promoting apoptosis of keratinocytes, partially by activating the cAMP/PKA signaling pathway.

## Introduction

Psoriasis is a chronic, recurrent, immune-mediated inflammatory disease that affects approximately 1%–5% of the population worldwide. The characteristic skin lesions of psoriasis are erythema and scaly, which can cause itching, and the lesions are often located at the elbows, knees and even extend to the limbs and scalp (Armstrong and Read, 2020).

Keratinocytes are not only involved in the initial onset stage of psoriasis but also contribute to the maintenance of the chronic stage (Malakou et al., 2018). The homeostasis between the proliferation and differentiation in keratinocytes is disrupted, resulting in the formation of a self-amplifying cycle (Sestito et al., 2011; Albanesi et al., 2018). Increased resistance to apoptosis has also been observed in activated keratinocytes (Janjetovic et al., 2014). Several anti-psoriatic therapies, such as Vitamin D3 analogs (Kim and Frampton, 2016) and photochemotherapy (Morita, 2018), are effective in treating psoriasis *via* the normalization of keratinocyte proliferation and differentiation. Therefore, regulating the proliferation, differentiation, and apoptosis in keratinocytes may be a therapeutic target for psoriasis.

Cyclic adenosine monophosphate (cAMP) is an important second messenger utilized for the regulation of keratinocyte proliferation and differentiation. Increased levels of cAMP are associated with inhibiting the proliferation of epidermal cells (Delescluse et al., 1974; Boyce and Ham, 1983; Yamanishi et al., 1989). Human keratinocytes express high levels of β_2_ adrenergic receptors (β_2_-AR). In keratinocytes, stimulation of β_2_-AR results in an adenylate cyclase (AC)-mediated increase in cAMP and an increase in intracellular [Ca^2+^] *via* cAMP-dependent and independent pathways (Orenberg et al., 1983). Increases in intracellular [Ca^2+^] inhibit keratinocyte proliferation but promote keratinocyte differentiation (Mammone et al., 1998). Epidermal cells in psoriatic skin lesions have decreased β-Adrenergic responsiveness and enhanced keratinocyte proliferation (Eedy et al., 1990; Takahashi et al., 1996). In addition, β-AR antagonists are the most common drugs to induce or aggravate psoriasis (Wolf et al., 1994; Basavaraj et al., 2010). Therefore, we hypothesized that topical administration of salmeterol (Sal), a novel selective long-acting β_2_-AR agonist, may be able to treat psoriasis. Therefore, in the present study, we observed the effects of salmeterol on the proliferation and apoptosis of keratinocytes and imiquimod (IMQ)-induced psoriasis-like dermatitis in mice.

Phosphodiesterase (PDE) terminate the actions of cAMP by mediating its hydrolysis to AMP. β_2_-AR agonists induce high levels of cAMP through a G protein-coupled receptor mechanism, while PDE4 inhibitors increase intracellular cAMP concentration by inhibiting cAMP hydrolysis. We hypothesized that the complementary action for both compounds may result in increased or sustained levels of cAMP. It has been reported that the combination of roflumilast and indacaterol, a long-acting β_2_-AR agonist, exerts synergistic anti-inflammatory and anti-fibrotic effects in human lung fibroblasts (Tannheimer et al., 2012). Therefore, we investigated whether salmeterol combined with roflumilast has a synergistic effect on improving psoriasis-like skin lesions.

## Materials and Methods

### Regents

Salmeterol (Sal), roflumilast (Rof), and extracellular signal-regulated kinase (ERK) inhibitor U0126 were obtained from Selleck (Shanghai, China). PKA agonist forskolin (FSK) and PKA inhibitor H89 were purchased from LC Laboratories. Tumor necrosis factor-α (TNF-α) was synthesized in the PeproTech (Cranbury, NJ, United States).

### Animals

Female C57BL/6J mice (7–8 weeks old) were purchased from Sipeifu Biotechnology (Beijing, China). They were fed with water and food *ad libitum* at 23 ± 2°C in a 12-h light/dark cycle. Animal experiments were approved by the Animal Ethics Committee of Tongji Medical College, Huazhong University of Science and Technology. The investigation conforms to the Guide for the Care and Use of Laboratory Animals published by the US National Institutes of Health (NIH Publication No. 85-23, revised 1985). We followed ARRIVE guidelines when reporting this study.

### Cell Culture

Human keratinocyte cell line (HaCaT cell line) was purchased from the China Center for Type Culture Collection (Wuhan, China). The HaCaT cells were cultured in Dulbecco’s modified Eagle’s medium (DMEM, HyClone, United States) containing 10% fetal bovine serum (FBS) and 1% penicillin and streptomycin in a humidified incubator with 5% CO_2_ at 37°C.

### Cell Viability Assay

Cell viability was analyzed with Cell counting kit-8 (CCK-8, Beijing Labgic Technology, Beijing, China). Briefly, cells were seeded in 96-well plates. After the indicated treatment, CCK-8 was added and incubated for 4 h. Absorbance was assessed at 450 nm with a microplate reader (Awareness Technology, Inc., United States).

### Cell Proliferation Assay

Cell proliferation assay is based on incorporating 5-ethynyl-2′-deoxyuridine (EdU) into genomic DNA, which was analyzed using Cell-Light EdU Apollo567 *In Vitro* Kit (Riobio, Guangzhou, China). Apollo staining and Hoechst 33342 staining (for nuclear staining) were performed. Fluorescence images were analyzed using ImageJ (NIH, Bethesda, MD, United States). The EdU incorporation rate was equal to the ratio of EdU-positive cells (red)/total number of Hoechst-positive cells (blue).

### Fluorescence Resonance Energy Transfer Measurements

HEK293 cells expressing nuclear-specific PKA biosensor NLS-AKAR3 were rinsed and maintained in PBS for fluorescence resonance energy transfer (FRET) as described previously (Liu et al., 2012). FRET was recorded by exciting the donor fluorophore at 405–455 nm and measuring emission fluorescence with two filters (470DF30 for cyan and 535DF30 for yellow). The acquisition was set with 200-ms exposure in both channels and 20-s elapses. Images in both channels were subjected to background subtraction, and ratios of yellow-to-cyan color were calculated at different time points. The donor/acceptor FRET ratio was calculated and normalized to the ratio value of baseline.

### IMQ-Induced Psoriasis-Like Dermatitis Mouse Model

**FIGURE 1:**
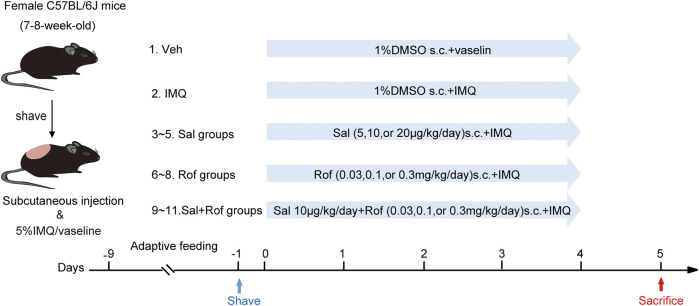
Pharmacological intervention of psoriasis-like dermatitis induced by IMQ in mice. Experimental scheme of psoriasis-like dermatitis induced by IMQ in mice. Sal, Rof, and the combination of Sal and Rof were subcutaneously administered under the skin for 5 days.

After 1 week of adaptive feeding, hair was removed from the back regions of mice 24 h before treatment (Figure 1). The mice were randomly assigned into the following 11 groups (*n* = 5–8 mice per group): vehicle group; IMQ group; Sal therapy groups (5, 10, or 20 μg/kg/d); Rof therapy groups (0.03, 0.1, or 0.3 mg/kg/d); and Sal (10 μg/kg/d) + Rof (0.03, 0.1, or 0.3 mg/kg/d) combination groups. The shaved back skin of the vehicle group mice and IMQ group mice received subcutaneously injections at five sites with the same amount of 1% dimethyl sulfoxide (DMSO; Sigma, United States). The vehicle group mice received topical application of Vaseline jelly, while the other mice received a daily topical dose of 62.5 mg of 5% IMQ cream on the shaved back skin for five consecutive days, 4 h after the subcutaneous injection. The severity of psoriasis-like lesions was scored based on the clinical Psoriasis Area and Severity Index (PASI). On day 6, the mice were sacrificed, and skin tissue was taken for further experiments.

### Histology and Immunohistochemistry Staining

Skin tissue was freshly harvested, fixed in 4% paraformaldehyde solution, and embedded in paraffin. The tissue sections (3 μm) were stained with hematoxylin and eosin (H&E) or incubated with anti-Ki67 (Servicebio, Wuhan, China). Images were captured using a light microscope, and analyzed using ImageJ. The mean thickness value was calculated by three to five random fields of view in every section. Cells positive for Ki67 in every section were counted and expressed as positive cells per mm length of basement membrane.

### TUNEL Assay

Terminal deoxynucleotidyl transferase dUTP nick-end labeling (TUNEL) assay was performed on skin sections and HaCaT cells using the One Step TUNEL Apoptosis Assay Kit (Beyotime Biotechnology, China) according to the manufacturer’s instruction. Fluorescence images were visualized with fluorescence microscope (Olymbus, Japan) and randomly selected three to four fields were analyzed by ImageJ. The TUNEL positive rate was equal to the ratio of TUNEL-positive cells (red)/total number of DAPI-positive cells (blue).

### Western Blotting Analysis

Skin tissues and cells were lysed with the RIPA lysis buffer (Servicebio, Wuhan, China) containing protease and phosphatase inhibitor cocktail (Roche, Basel, Switzerland). The protein concentration was measured by BCA assay (Beyotime Biotechnology, Shanghai, China). Lysates (20–40 μg total protein) were resolved by SDS-PAGE. The extracts were then transferred onto a PVDF membrane (Merck Millipore, Billerica, MA). The following primary antibodies were used: pPDE4D (1:500; Abcam, Cambridge, UK); pCREB (1:500), ERK (1:1,000) and phospho-ERK (pERK, 1:500) from Cell Signaling Technology (Danvers, MA, United States); CREB (1:1,000) and Bax (1:1,000) from Bimake (Houston, TX, United States); K17 (1:500; Santa Cruz Biotechnology, United States); PDE4D (1:500), Bcl2 (1:200), and GAPDH (1:10,000) from Proteintech (Chicago, IL, United States). Chemiluminescent detection was performed with horseradish peroxidase–coupled secondary antibody from Proteintech (Chicago, IL, United States) and an ECL chemiluminescence reagent kit (Beijing Labgic Technology, China). Band densities were quantified using ImageJ software.

### Cyclic AMP Content Assay

Cyclic AMP content in skin sections were measured with the cAMP-Glo™ Assay kit (Promega, Madison, WI) following the manufacture’s instruction.

### Statistical Analysis

Data were presented as mean ± standard error of the mean (SEM). The sample size for each group is shown in the figure legends. At least three sets of independent experiments were performed in the *in-vitro* studies. Data were analyzed by unpaired *t* test, one-way ANOVA followed by Turkey’s post-hoc test, or two-way ANOVA followed by Turkey’s multiple comparisons test. *p* < 0.05 was considered statistically significant. Statistical analysis was performed with GraphPad Prism8.0 (GraphPad Software, Inc., San Diego, CA, United States).

## Results

### Salmeterol Suppresses Cell Growth and Increases Cell Apoptosis

**FIGURE 2:**
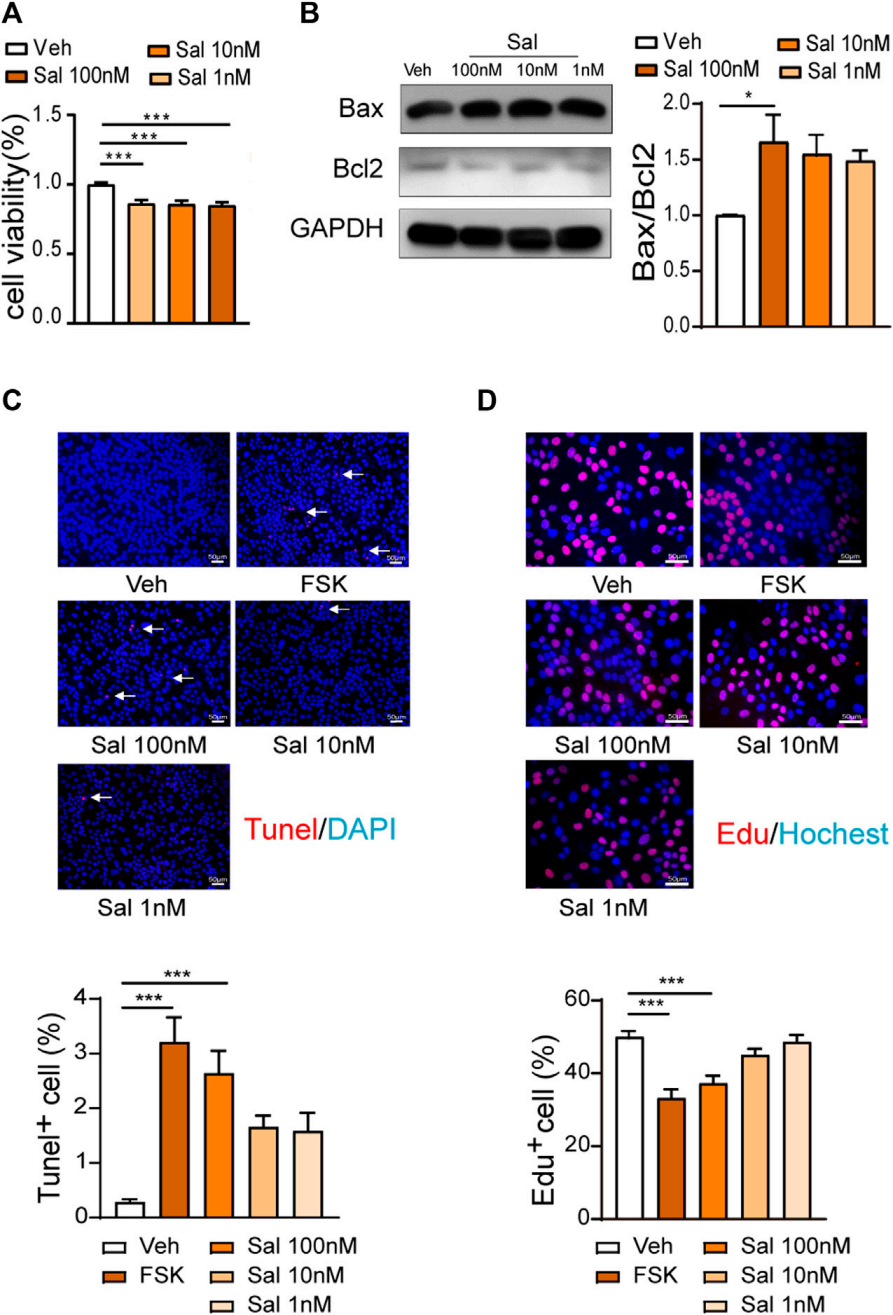
Effect of Salmeterol on cell viability, cell proliferation and apoptosis. **(A,B)** HaCaT cells were cultured in DMEM containing 10% FBS and treated with Sal at the indicated concentration for 48 h. **(A)** Cell viability was measured by a CCK8 assay (*n* = 3). **(B)** Western blotting analyses of the Bax/Bcl2 ratio (*n* = 4). **(C,D)** HaCaT cells were cultured in DMEM containing 10% FBS and then treated with Sal at the indicated concentrations or FSK (10 μM) for 48 h (*n* = 3). **(C)** Cell apoptosis was measured by a TUNEL assay (×200, Scale bar = 50 μm). **(D)** Cell proliferation was measured by EdU incorporation (×400, Scale bar = 50 μm). Data are presented as the mean ± SEM. *p < 0.05 and ***p < 0.001 by one-way ANOVA followed by Tukey’s post-hoc test.

Salmeterol inhibited cell viability in serum culture conditions, but there was no difference among various concentrations (Figure 2A). Compared to the vehicle group, 100 M salmeterol upregulated the ratio of Bax and Bcl2 (Figure 2B). The TUNEL and EdU incorporation assays showed that salmeterol (100 nM) resulted in a significant decrease in proliferation and increase in apoptosis. Interestingly, forskolin (10 μM), a PKA agonist, induced an effect similar to that of salmeterol (Figures 2C,D). These data suggested that the effect of salmeterol on cell proliferation and apoptosis may be related to PKA signaling.

### Salmeterol-Induced cAMP/PKA Activation Influences Cell Proliferation and Apoptosis in HaCaT Cells Under TNF-α Stimulation

**FIGURE 3:**
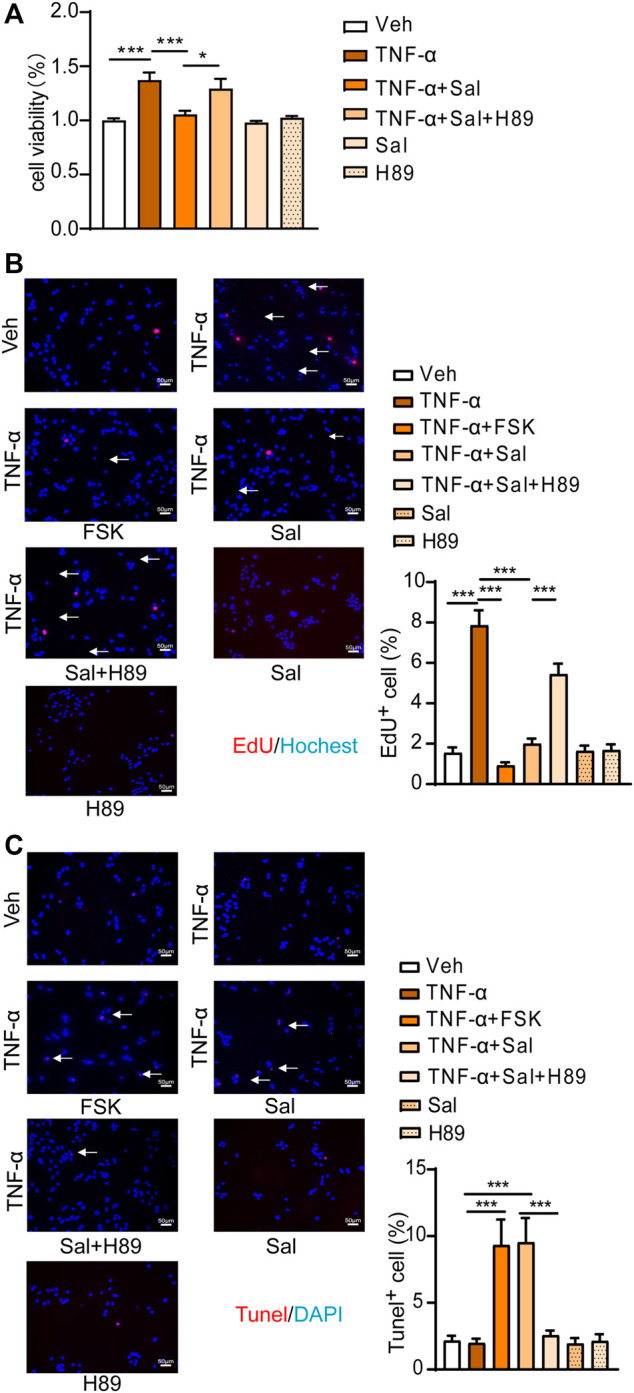
Salmeterol regulates TNF-α-stimulated cell proliferation and the apoptosis associated with activation of the cAMP/PKA pathway. HaCaT cells were serum starved overnight. During the experiment, HaCaT cells were cultured without serum. HaCaT cells were pretreated with the PKA inhibitor, H89 (1 μM), for 30 min followed by treatment with Sal (100 nM). After 1 h, HaCaT cells were exposed to TNF-α (10 ng/ml) for 48 h (*n* = 3). **(A)** Cell viability was measured by a CCK8 assay. **(B)** Cell proliferation was measured by EdU incorporation (×200, Scale bar = 50 μm). **(C)** Cell apoptosis was measured by a TUNEL assay (×200, Scale bar = 50 μm). Data are presented as the mean ± SEM. *p < 0.05 and ***p < 0.001 by one-way ANOVA followed by Tukey’s post-hoc test.

Inflammatory factors such as TNF-α, IL-23, and IL-17 contribute to the pathogenesis of psoriasis (Lowes et al., 2014)**.** We further tested the effect of salmeterol on cell proliferation and apoptosis under TNF-α stimulation. Treatment of HaCaT cells with TNF-α resulted in significant increases in cell viability (Figure 3A) and cell proliferation (Figure 3B), and these effects were inhibited by forskolin (PKA agonist) or salmeterol. The inhibitory effect of salmeterol on TNF-α-induced hyperproliferation was attenuated by the PKA inhibitor, H89, indicating that activation of cAMP/PKA pathway contributes to the influence of salmeterol on TNF-α induced cell proliferation (Figure 3B). Under TNF-α stimulation, salmeterol or forskolin promoted cell apoptosis, which played a part in the inhibition of TNF-α-induced cell viability (Figure 3C). H89, a PKA inhibitor, also reversed this effect of salmeterol, suggesting that salmeterol-activated cAMP/PKA signaling is involved in the promotion of cell apoptosis.

### Salmeterol Activates the PKA/CREB Signaling Pathway

**FIGURE 4:**
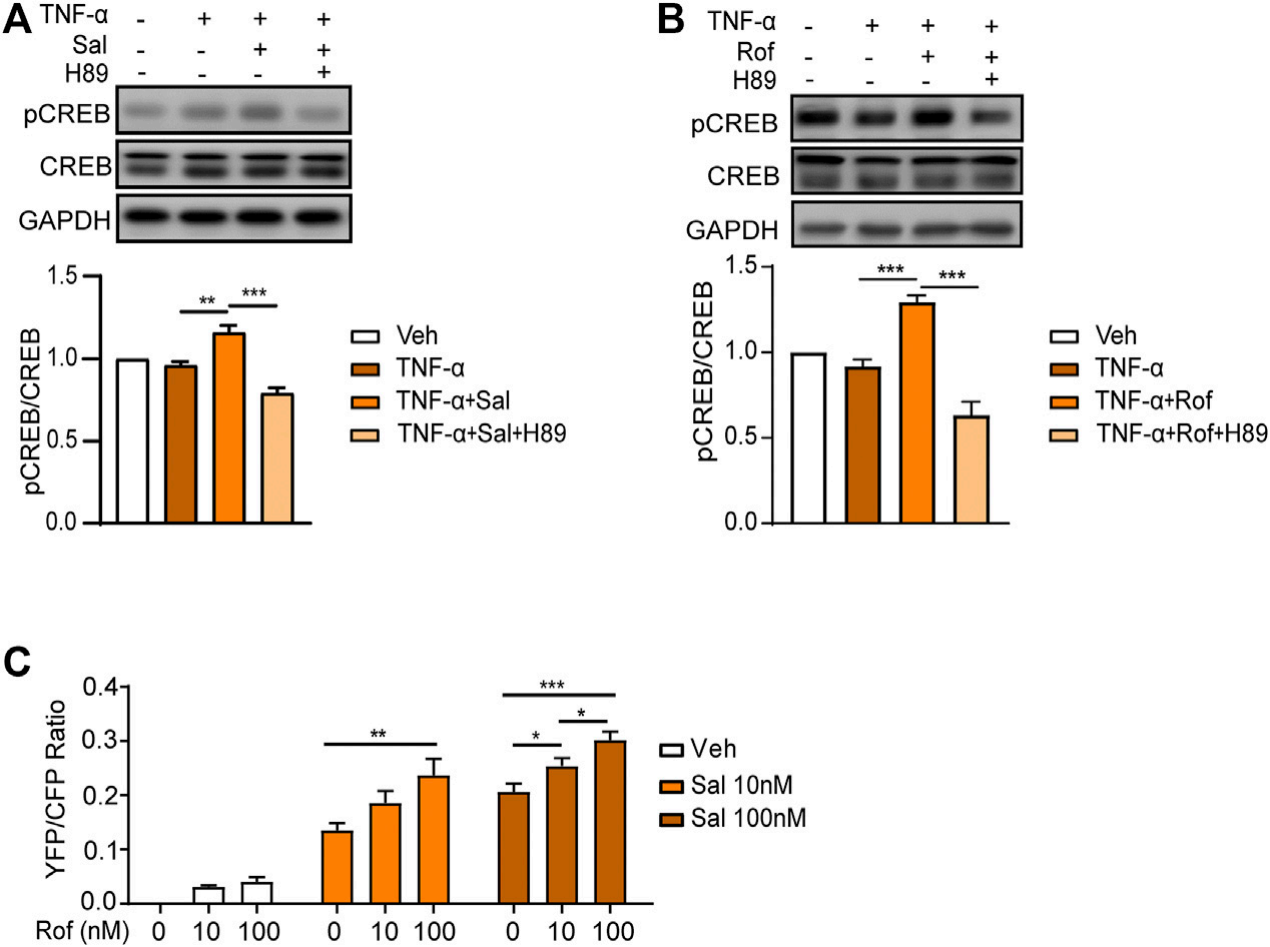
Salmeterol and roflumilast induce PKA/CREB activity. **(A,B)** HaCaT cells were serum starved overnight. During the experiment, HaCaT cells were cultured without serum. The cells were pretreated with the PKA inhibitor, H89 (1 μM), for 30 min followed by treatment with Sal (100 nM, 1 h) or Rof (100 nM, 30 min). The cells were then treated with TNF-α (10 ng/ml) for 48 h. Representative images and quantification of Western blots for phospho-CREB (pCREB), CREB, and GAPDH (*n* = 5). **(C)** PKA activity was measured by a FRET assay. HEK293 cells expressing the nuclear-specific PKA biosensor, NLS-AKAR3, were treated with Sal or Rof at the indicated concentration. The changes in FRET ratio of the PKA biosensor were recorded, and the maximal responses were plotted (*n* = 11–35). Data are presented as the mean ± SEM. **(A,B)** **p < 0.01 and ***p < 0.001 by one-way ANOVA followed by Tukey’s post-hoc test. **(C)** *p < 0.05, **p < 0.01, and ***p < 0.001 by two-way ANOVA followed by Tukey’s multiple comparisons test.

A PDE4 inhibitor has been approved for the treatment of moderate-to-severe plaque psoriasis and psoriatic arthritis (Papp et al., 2013). The anti-inflammatory effect of PDE4 inhibitors is associated with the enhancement of cAMP-dependent PKA-CREB signaling (Jin et al., 2005; Serezani et al., 2008; Milakovic and Gooderham, 2021). As shown in Figure 4A, salmeterol increased phosphorylation of CREB in HaCaT cells exposed to TNF-α, which was attenuated by the PKA inhibitor, H89. The effect of salmeterol on PKA-CREB signaling was similar to that of roflumilast (Figure 4B). Taken together, these results indicated that salmeterol and roflumilast activated the cAMP/PKA/CREB pathway. By FRET assay (Figure 4C), we also determined that salmeterol and roflumilast activated the cAMP/PKA signaling pathway in a dose-dependent manner. Notably, the combination of salmeterol and roflumilast synergistically increased PKA activity.

### Salmeterol Ameliorates IMQ-Induced Psoriasis-Like Skin Lesion

**FIGURE 5:**
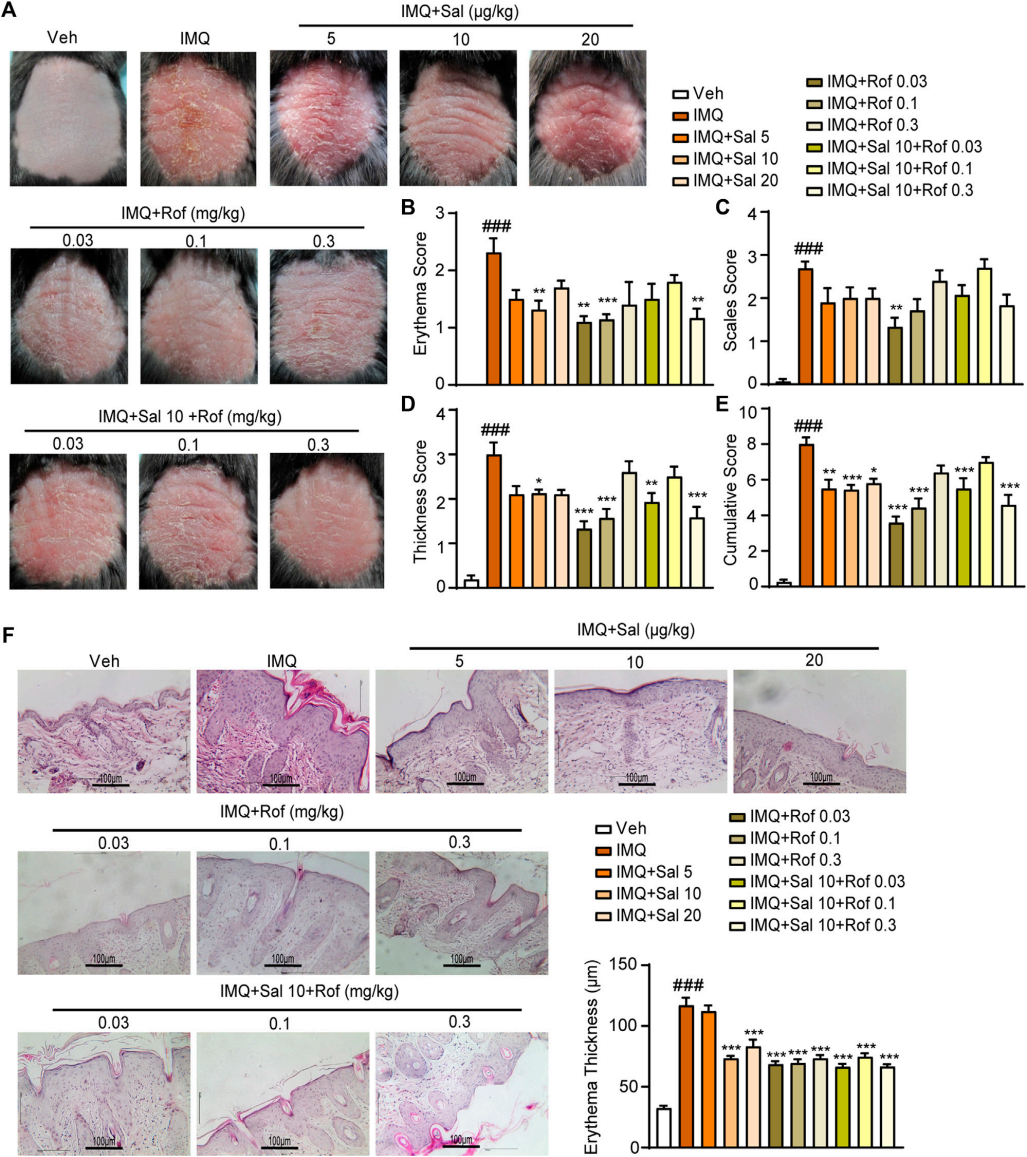
Effects of salmeterol, roflumilast, and salmeterol combined with roflumilast on psoriasis-like dermatitis induced by IMQ in mice. Psoriasis-like dermatitis was induced by IMQ in mice. Sal, Rof, and the combination of Sal and Rof were subcutaneously administered under the skin for 5 days. **(A)** Macroscopic presentation of mice on the sixth day after IMQ treatment. **(B–E)** Quantitative severity assessment on the sixth day after IMQ treatment (*n* = 5–8). Erythema **(B)**, scales **(C)**, and thickness **(D)** of the back skin were scored on a scale from 0 to 4. The cumulative score **(E)** was calculated. **(F)** Representative H&E staining of cross-sectional slices of the back skin on the sixth day after IMQ treatment. The epidermal thickness of the dorsal skin was measured by five randomly selected fields per section of each mouse. (*n* = 5–8; ×200, Scale bar = 100 μm). Data are presented as the mean ± SEM. ###*p* < 0.001 vs. vehicle group; *p < 0.05, **p < 0.01, and ***p < 0.001 vs. IMQ-treated group by one-way ANOVA followed by Tukey’s post-hoc test.

To analyze the potential therapeutic effect of topical administration of salmeterol on psoriasis *in vivo*, we constructed an IMQ-induced mouse model that effectively replicated the characteristics of psoriatic lesions in humans (van der Fits et al., 2009) to investigate whether salmeterol in combination with roflumilast has a synergistic effect. On the fifth day, the IMQ-treated mice showed thickened, erythematous, and scaly back skin compared to the vehicle group mice, reflecting psoriasis-like skin conditions (Figure 5A), and the IMQ-treated mice showed significant increases in lesion severity scores (Figures 5B–D). Histopathological analysis of back skin with hematoxylin and eosin staining showed that IMQ induced epidermal thickening (Figure 5F). The above results demonstrated that we successfully constructed an IMQ-induced psoriasis-like mouse model. The phenotypical changes of the back skin (Figure 5A) and lesion severity score (Figures 5B–D) showed that topical treatment with salmeterol, roflumilast, or combination of salmeterol and roflumilast significantly attenuated IMQ-induced psoriatic traits and reduced IMQ-induced epidermal thickening (Figure 5F). However, there was no significant difference with the combination treatment compared to treatment with either salmeterol or roflumilast alone.

### Salmeterol Effectively Reduces Epidermal Hyperproliferation and Promotes Apoptosis Associated With PKA/CREB Activation in IMQ-Induced Psoriasis-Like Mouse Skin

**FIGURE 6:**
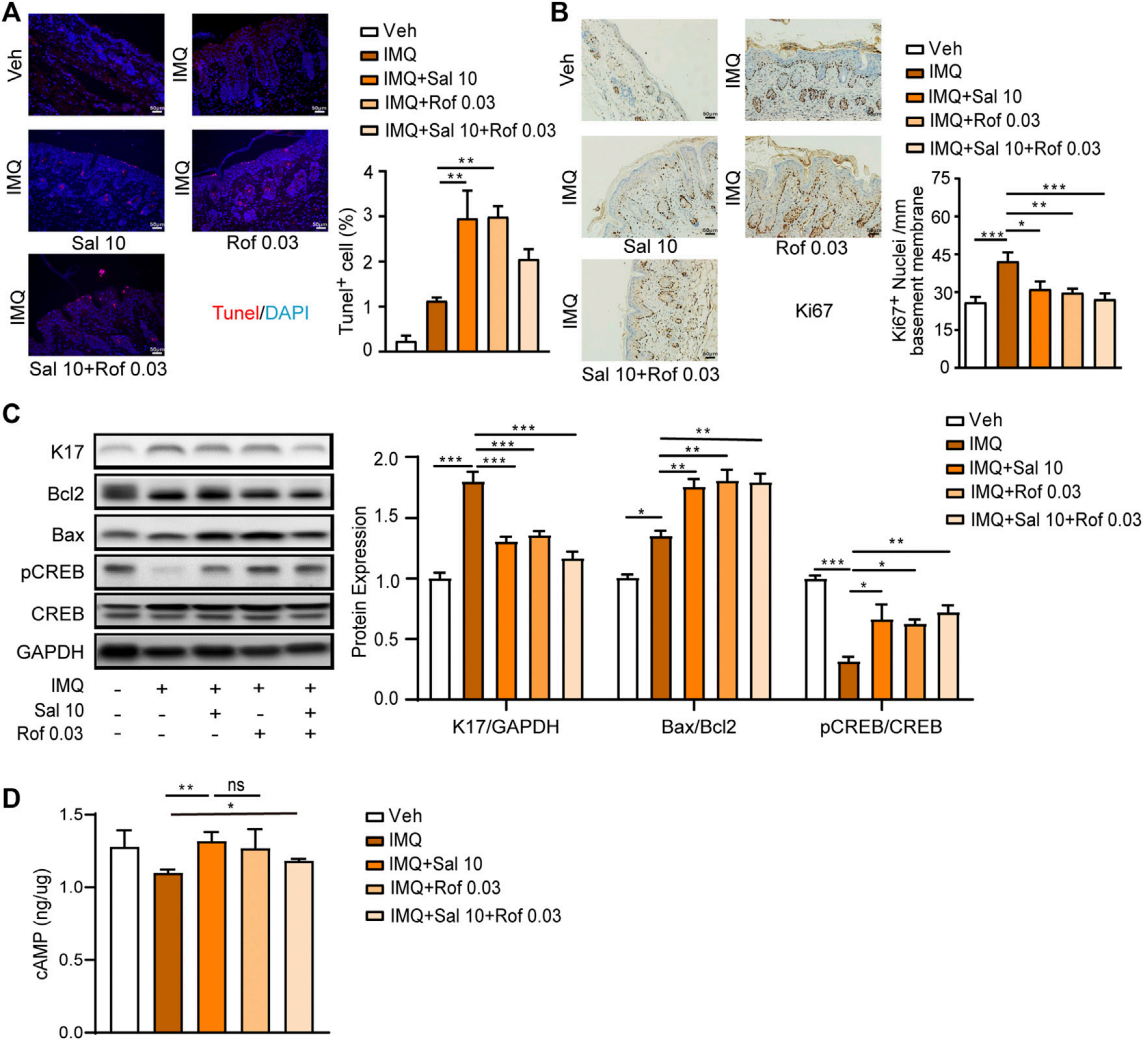
Effects of salmeterol, roflumilast, and salmeterol combined with roflumilast on cell proliferation and apoptosis in the epidermis of mice with IMQ-induced psoriasis. Psoriasis-like dermatitis was induced by IMQ in mice. Sal, Rof, and the combination of Sal and Rof were subcutaneously administered under the skin for 5 days. **(A)** Representative TUNEL staining of cross-sectional sections of the back skin of mice on the sixth day (*n* = 3; ×200, Scale bar = 50 μm). **(B)** Representative Ki67 staining of cross-sectional sections of the back skin of mice on the sixth day (*n* = 3; ×200, Scale bar = 50 μm). **(C)** Expression levels of K17, Bcl2, Bax, pCREB, and CREB in the back skin were detected by Western blotting. GAPDH served as the loading control (*n* = 4). **(D)** cAMP levels were detected in skin tissues (*n* = 4–7). Data are presented as the mean ± SEM. **(A–C)** *p < 0.05, **p < 0.01, and ***p < 0.001 by one-way ANOVA followed by Tukey’s post-hoc test. **(D)** *p < 0.05 and **p < 0.01by unpaired t test. ns, not significant.

Based on the cumulative score (Figure 5E) and the improvement of epidermal thickness (Figure 5F), mouse skin tissues obtained from the Sal (10 μg/kg) group, Rof (0.03 mg/kg) group or Sal (10 μg/kg) + Rof (0.03 mg/kg) group were used to analyze cell apoptosis, cell proliferation, and cAMP levels. The TUNEL assay showed that compared to the IMQ-treated group, salmeterol treatment and roflumilast treatment significantly promoted cell apoptosis in the epidermis (Figure 6A) and increased the Bax/Bcl2 ratio (Figure 6C). The combination treatment also increased the Bax/Bcl2 ratio but did not result in a statistically significant increase in TUNEL-positive cells (Figure 6A). As assessed by immunohistochemistry staining, IMQ treatment increased the expression of Ki67 in the epidermis, whereas salmeterol, roflumilast, and the combination treatment inhibited the IMQ-induced Ki67 expression (Figure 6B). Consistently, IMQ treatment significantly increased the expression of the epidermal proliferation marker, keratin 17 (K17), whereas salmeterol, roflumilast, and the combination treatment inhibited the IMQ-induced K17 expression (Figure 6C). Consistent with the above results, there was no significant difference in the combination treatment compared to treatment with salmeterol or roflumilast alone.

We further determined the effect of salmeterol on PKA/CREB activation in IMQ-induced psoriasis-like skin lesions by Western blot analysis. CREB phosphorylation was inhibited by IMQ treatment, while salmeterol, roflumilast, and the combination treatment partly reversed the IMQ-inhibited CREB phosphorylation. However, the effect of the combination treatment on CREB phosphorylation was not significantly different compared to treatment with salmeterol or roflumilast alone (Figure 6C). Next, we explored whether the level of CREB phosphorylation is associated with cAMP levels. As shown in Figure 6D, the cAMP levels were slightly decreased in skin tissue after IMQ treatment, but the topical treatments showed higher cAMP levels with no synergistic effect in the combination treatment group. These results partly explained why there was no synergistic effect of the combination treatment *in vivo*. Thus, these results suggested that salmeterol ameliorates IMQ-induced psoriasis-like skin lesions by inducing apoptosis and negatively regulating proliferation *via* activating CREB.

### ERK Activation in the Combination Treatment Mediates the Lack of Synergisms

**FIGURE 7:**
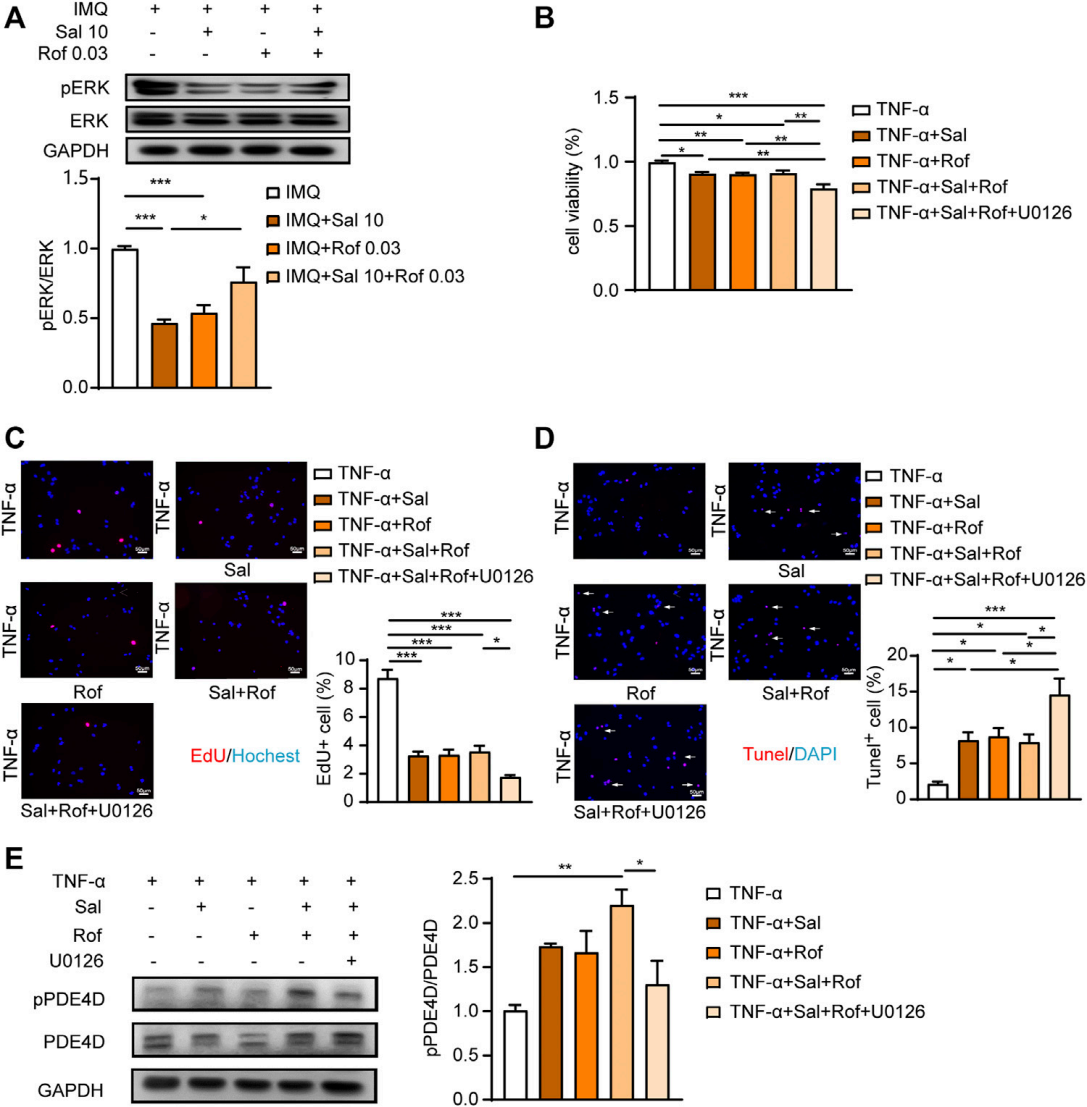
ERK activation in the combination treatment mediates the lack of synergisms. **(A)** Psoriasis-like dermatitis was induced by IMQ in mice. Sal, Rof, and the combination of Sal and Rof were subcutaneously administered under the skin for 5 days. Expression levels of pERK and ERK in the back skin were detected by Western blotting. GAPDH served as the loading control (*n* = 4). **(B–D)** HaCaT cells were serum starved overnight. During the experiment, HaCaT cells were cultured without serum. HaCaT cells were pretreated with U0126 (1 μM), for 30 min followed by treatment with Sal (100 nM, 1 h) or Rof (100 nM, 30 min). The cells were then exposed to TNF-α (10 ng/ml) for 48 h (*n* = 3). **(B)** Cell viability was measured by a CCK8 assay. **(C)** Cell proliferation was measured by EdU incorporation (×200, Scale bar = 50 μm). **(D)** Cell apoptosis was measured by a TUNEL assay (×200, Scale bar = 50 μm). **(E)** HaCaT cells were serum starved overnight. During the experiment, HaCaT cells were cultured without serum. HaCaT cells were pretreated with U0126 (1 μM), for 30 min followed by treatment with Sal (100 nM, 1 h) or Rof (100 nM, 30 min). The cells were then exposed to TNF-α (10 ng/ml) for 1 h (*n* = 3). Expression levels of pPDE4D and PDE4D were detected by Western blotting. GAPDH served as the loading control. Data are presented as the mean ± SEM. *p < 0.05, **p < 0.01, and ***p < 0.001 by one-way ANOVA followed by Tukey’s post-hoc test.

ERK pathway is a well-known regulator of cell growth, proliferation, and apoptosis. Several studies suggest the association between activation of ERK signaling and psoriasis development (Takahashi et al., 2002; Johansen et al., 2005). The inhibition of ERK relieved skin symptoms in a mouse imiquimod-induced model (Wu et al., 2018; Huang X et al., 2019). A latest study reported that β_2_-AR agonists promote ERK dephosphorylation in human airway epithelial cells by cAMP-PKA signaling (Hamed et al., 2021), although some earlier studies showed that PKA mediates the switch of coupling of β_2_-AR from Gs to Gi and initiate the activation of ERK pathway (Daaka et al., 1997; Zamah et al., 2002; Smith et al., 2021). Thus, we examined ERK phosphorylation in psoriasis-like skin lesions. As shown in Figure 7A, both salmeterol and roflumilast alone group showed ERK dephosphorylation compared to the IMQ-treated group, but the combination treatment promoted ERK phosphorylation compared to salmeterol or roflumilast treatment alone. We further found that the synergistic effects of anti-proliferation and induction of apoptosis appeared in the presence of ERK inhibitor U0126 (Figures 7B–D).

As shown in Figure 7E, both salmeterol and roflumilast alone group slightly increased the level of PDE4D phosphorylation, which is consistent with previous studies that PDE4s are activated by PKA phosphorylation, providing a negative feedback mechanism by which cAMP regulates its own level (Sette and Conti, 1996; Rochais et al., 2004). In addition, the combination treatment significantly increased the level of PDE4D phosphorylation (Figure 7E), which could partly explain that there was no synergistic effect on increasing cAMP level in the combination treatment group (Figure 6D). Of note, ERK inhibitor blocked the combination treatment-increased PDE4D phosphorylation (Figure 7E), which is consistent with previous studies that PDE4D activity is regulated by extracellular-signal regulated kinases (Wang et al., 2017; Xu et al., 2020).

## Discussion

Psoriasis is a common immune-mediated inflammatory disease. However, keratinocytes play a critical role in inducing the early pathogenic events and sustaining the prolonged phase of the disorder (Ni and Lai, 2020). The keratinocytes in psoriatic lesions have an abnormal proliferation and differentiation process, and they are resistance to apoptosis (Sestito et al., 2011; Janjetovic et al., 2014). Hence, inhibiting proliferation or promoting apoptosis in keratinocytes is a feasible approach to mitigating psoriatic lesions (Kim and Frampton, 2016; Morita, 2018; Huang T. H et al., 2019). Herein, we found that salmeterol ameliorated the psoriasis-like lesions induced by IMQ in mice. Mechanistically, this protective effect was associated with the inhibition of proliferation and the promotion of apoptosis in keratinocytes, and the cAMP-dependent PKA/CREB pathway may play a role in this protective effect.

Previous studies have shown that intracellular cAMP regulate keratinocyte proliferation (Delescluse et al., 1974; Yamanishi et al., 1989), whereas catecholamines increase intracellular cAMP levels and inhibit cell proliferation (Simard et al., 2020). β_2_-AR, which can be functionally coupled with AC to promote cAMP production, is highly expressed on keratinocytes, but psoriatic keratinocytes are less responsive to β-AR agonists (Eedy et al., 1990), resulting in an attenuated cAMP increase response. A recent study has reported that topical salbutamol, a short-acting β_2_-AR agonist, improves IMQ-induced psoriatic lesions in mice (Liu et al., 2020). These results suggest that activation of the β_2_-AR/cAMP signaling pathway may be a therapeutic approach for psoriasis. In the present study, we showed that salmeterol, a long-acting β_2_-AR agonist, improved IMQ-induced psoriatic lesions *via* subcutaneous administration. Through FRET assay, we observed that salmeterol significantly increased PKA activity and increased levels of pCREB, which were reversed by PKA inhibitor H89. Similar to forskolin, a PKA activator, salmeterol inhibited proliferation and promoted apoptosis in HaCaT cells under serum culture or exposure to inflammatory factors, and the PKA inhibitor, H89, reversed these effects. Salmeterol partially inhibited the pCREB/CREB downregulation induced by IMQ in IMQ-induced psoriatic lesions. These results demonstrated that salmeterol regulates proliferation and apoptosis, at least in part, by activating cAMP-dependent PKA/CREB signaling, suggesting that salmeterol may have potential to treat psoriasis.

PDE4 controls the amplitude and duration of the cAMP signal through hydrolysis to 5′-AMP. Oral apremilast was the first available PDE4 inhibitor approved for the treatment of moderate-to-severe plaque psoriasis and psoriatic arthritis (Papp et al., 2013). A Phase 2b, parallel-group, double-blinded, vehicle-controlled trial has been conducted to evaluate the safety and efficacy of once-daily topical roflumilast in plaque psoriasis (Lebwohl et al., 2020; Mahil and Smith, 2020).

The enhancement of cAMP-dependent PKA-CREB signaling is associated with the anti-inflammatory effect of PDE4 inhibitors (Jin et al., 2005; Serezani et al., 2008). Moreover, PDE4 inhibitors synergistically inhibit the release of pro-inflammatory and pro-fibrotic mediators caused by TGF-β2 when combined with the long-acting β_2_-AR agonist (Tannheimer et al., 2012); the combination of a PDE4 inhibitor and forskolin have additive inhibition on LPS-induced proinflammatory factors release in RAW264.7 cells (Li et al., 2020), and its mechanism may be related to the further enhancement of PKA/CREB signaling. We observed that the combination of salmeterol and roflumilast further enhanced PKA activity *in vitro* through FRET-based sensors; we also tested the synergistic effect of roflumilast and salmeterol in IMQ-induced psoriatic lesions. However, the synergistic effect in improving psoriatic lesions in mice was not observed with the combination treatment, and the effect even tended to decrease when the combination included a high dose of roflumilast. Moreover, the combination treatment had no synergistic effect in inhibiting the reduction of CREB phosphorylation and cAMP contents induced by IMQ in skin lesions.

β_2_-AR is a prototypical G protein-coupled receptor (GPCR). Besides the G_s_/cAMP-dependent PKA signaling pathway, β_2_-AR also can activate G_i_ protein-dependent pathways. PKA mediates the switch of coupling from G_s_ to G_i_ and initiate a new set of signaling events including the activation of ERK pathway (Daaka et al., 1997; Zamah et al., 2002; Smith et al., 2021). Studies revealed that activated β_2_-AR induces PKA activity at a broad range of agonist concentrations. However, β_2_-AR/G_i_ coupling occurs only at a saturated concentration of agonist (Liu et al., 2009). PKA inhibitor blocked β_2_-AR/G_i_ coupling and subsequent activation of MAPK (Daaka et al., 1997). The combination treatment promoted ERK phosphorylation in psoriasis-like skin lesions compared to salmeterol or roflumilast treatment alone. The observed increase in ERK phosphorylation may be due to the switch in the coupling of β_2_-AR from G_s_ to G_i_ through further activated PKA induced by the combination treatment. Furthermore, the synergistic effects of anti-proliferation and induction of apoptosis appeared in the presence of ERK inhibitor U0126. Of note, ERK inhibitor blocked the combination treatment-increased PDE4D phosphorylation. These findings confirmed that activation of ERK pathway may be a reason for the lack of synergisms in combination of salmeterol and roflumilast. It was shown previously that the effect of salmeterol on cytokine transcription was not mediated by PKA, but could be completely blocked by inhibitors of either the ERK pathway (Tan et al., 2007). This would also explain why the synergistic effect of salmeterol and roflumilast combination was not observed since roflumilast-increased PKA might promote switch coupling of β_2_-AR from G_s_ to G_i_ and initiate ERK activation. Similar to ours, Tyrrell et al. reported that roflumilast increased the rate of airway-surface liquid (ASL) height recovery in cultures after cigarette smoke exposure compared with control. Interestingly, the clinically relevant combination of salmeterol and roflumilast induced an inhibition of the roflumilast effect, although both salmeterol and roflumilast are used to treat chronic obstructive pulmonary disease (COPD) (Tyrrell et al., 2015), suggesting that is a possibility that salmeterol affects the ability of roflumilast to inhibit PDE4. Together, we suggest that the coadministration of salmeterol and roflumilast cause a switch from β_2_-AR/G_s_ coupling to β_2_-AR/G_i_ coupling and subsequent ERK activation through further activated PKA induced by the combination treatmen. Increased ERK activity can lead on the one hand to induce cell proliferation and inhibit cell apoptosis, and on the other hand to increase PDE4D activation, leading to there was no therapeutic synergy between salmeterol and roflumilast in relieving psoriasis.

## Conclusion

Our study confirmed that salmeterol, a long-acting β_2_ adrenergic receptor agonist, improves IMQ-induced psoriasis-like skin lesions, which may be related to activation of the cAMP/PKA pathway, thereby inhibiting the proliferation of epidermal cells and promoting their apoptosis.

## Data Availability

The original contributions presented in the study are included in the article/Supplementary Material, further inquiries can be directed to the corresponding authors.

## Abbreviations

cAMP, cyclic adenosine monophosphate; CREB, cAMP response element-binding protein; DMEM, Dulbecco’s modified Eagle’s medium; EPAC1, exchange protein 1 activated by cAMP; ERK, extracellular signal-regulated kinase; EdU, 5-ethynyl-2′-deoxyuridine; FBS, fetal bovine serum; FRET, fluorescence resonance energy transfer; IMQ, imiquimod; β_2_-AR, β_2_ adrenergic receptor; PDE, phosphodiesterase; PKA, protein kinase A; Rof, roflumilast; Sal, salmeterol; TNF-α, tumor necrosis factor-α; TUNEL, terminal deoxynucleotidyl transferase dUTP nick-end labeling.
